# Making Sense of a Health Threat: Illness Representations, Coping, and Psychological Distress among *BRCA1/2* Mutation Carriers

**DOI:** 10.3390/genes12050741

**Published:** 2021-05-14

**Authors:** Hannah Brand, Dorothee Speiser, Laura Besch, Julia Roseman, Friederike Kendel

**Affiliations:** 1 Charité—Universitätsmedizin Berlin, Corporate Member of Freie Universität Berlin, Humboldt-Universität zu Berlin and Berlin Institute of Health, Gender in Medicine, Charitéplatz 1, 10117 Berlin, Germany; laura.besch@charite.de (L.B.); julia.roseman@charite.de (J.R.); friederike.kendel@charite.de (F.K.); 2 Charité—Universitätsmedizin Berlin, Corporate Member of Freie Universität Berlin, Humboldt-Universität zu Berlin and Berlin Institute of Health, Hereditary Breast and Ovarian Cancer Center, Charitéplatz 1, 10117 Berlin, Germany; dorothee.speiser@charite.de

**Keywords:** *BRCA1/2* mutation, genetic counseling, coping, illness representations, psychological distress

## Abstract

Little is known about how women with a *BRCA1/2* mutation develop an individual understanding of their breast and ovarian cancer risk and how this affects their psychological distress. In this study, we investigated associations between illness representations, coping strategies and psychological distress. *N* = 101 *BRCA1/2* mutation carriers answered self-report questionnaires on illness representations, coping strategies, cancer worry and depressive symptoms. Women without cancer were compared to women with a previous cancer diagnosis. Illness representations explained 50% and 45% of the variability in cancer worry and depressive symptoms, respectively. Woman perceiving severe consequences (*β* = 0.29, *p* < 0.01) and having more concerns (*β* = 0.37, *p* < 0.01) were found to report more cancer worry. Perceiving information about the mutation as less coherent (*β* = −0.17, *p* < 0.05) and experiencing negative emotional responses (*β* = 0.60, *p* < 0.01) were both associated with more depressive symptoms. Women with a previous cancer diagnosis show patterns of illness representations that are potentially more distressing than women without a cancer diagnosis. Findings suggest that physicians involved in counseling should pay attention to illness representations of distressed women. Thereby, it would be possible to detect maladaptive thoughts associated with the mutation, address negative emotions and encourage adaptive coping strategies.

## 1. Introduction

Women carrying a *BRCA1/2* mutation live with a substantially elevated lifetime risk of developing breast and ovarian cancer. Testing positive for a pathogenic *BRCA1* or *BRCA2* variant indicates a cumulative breast cancer risk, up to the age of 80, of 72% and 69%, respectively [1]. The pathogenic genetic variant also predisposes women to a cumulative risk for ovarian cancer up to 44% in *BRCA1*, and 17% in *BRCA2* mutation carriers [1]. Genetic tests identify these particularly vulnerable groups, allowing for more targeted interventions. For example, early detection programs and prophylactic interventions can contribute to reducing mortality and the risk of developing the disease [2,3]. Pathogenic BRCA variants confront mutation carriers with complex and potentially life-changing decisions regarding prevention strategies or early detection programs. Women show diverse attitudes and feelings in reaction to living with high cancer risks. Upon notification of the genetic result, some mutation carriers experience only a temporary increase in general and cancer-related distress or no distress at all [4,5]. Other women, however, report fear of developing cancer at a young age and passing the deleterious mutation on to their own children [6,7]. Recent systematic reviews mirror these heterogeneous results, with some studies indicating that carriers report higher distress than non-carriers [8,9,10], while others reveal reduced distress as a result of genetic counseling [9]. Consequently, there are *BRCA1/2* mutation carriers who display persistent heightened distress [11] and a “psychological response similar to the diagnosis of breast cancer itself” [12] (p. 588). 

### Common Sense Model of Self-Regulation of Health and Illness

Emotional and physical wellbeing depends not only on objective medical estimations, but also on subjective perceptions of a disease or health threat. Individuals vary markedly in these subjective interpretations of threats and illnesses: whereas one woman might consider the *BRCA1/2* gene mutation a manageable and treatable condition, another *BRCA1/2* mutation carrier might anticipate serious and long-lasting consequences for herself and her family. Leventhal’s Common Sense Model of Self-Regulation of Health and Illness (CSM) [13,14] describes how individuals develop their own “making sense” of an illness or health threat by forming their own illness representation, defined as an individual and subjective belief system regarding an illness. Illness representations develop from contact with diverse sources of information and evolve over time. In the case of *BRCA1/2* mutation carriers, the representation of the health threat (cancer) is mostly formed through direct experiences with family members, but is also influenced by friends, media and cultural beliefs [15]. The CSM posits distinct dimensions of illness representations: identity (labelling the illness), cause, timeline, consequences, coherence (understanding the illness), personal and treatment control and emotional representation (affective response, such as anger or grief) [16].

A meta-analysis evaluating the CSM in patients with cancer revealed specific patterns between illness representations and coping behavior, finding illness representations to be important determinants for adaptive outcomes in cancer [17]. The included studies consistently reported that perceiving more severe consequences was positively associated with passive and emotion-focused coping strategies, such as denial and cognitive reappraisal, and with maladaptive psychological health outcomes, such as anxiety and depression. By contrast, perceiving a higher level of control was positively related with problem-focused coping and greater psychological well-being. Negative emotional illness representations were strongly associated with denial coping strategies and negative psychological health outcomes, such as higher anxiety, depression, and psychological distress. In studies that focused exclusively on breast cancer patients, similar patterns of associations were found [17].

There are few studies that address illness representations, coping, and psychological distress in *BRCA1/2* mutation carriers. In one study, negative emotional representations predicted passive coping strategies and seeking social support, whereas anticipating severe consequences predicted both passive AND active coping strategies [18]. In terms of health outcomes, women with negative emotional representations and women who perceived breast cancer less coherently, that is understanding the disease’s pathology less thoroughly, displayed more anxiety and distress [18]. Perceiving the health threat to be more serious was associated with negative psychological health outcomes, such as cancer-related anxiety or distress [18,19] and more state anxiety [20]. 

**Figure 1:**
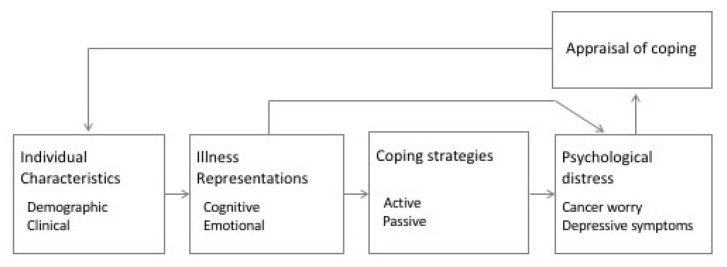
Conceptual model of predictors of psychological distress, adapted from Freemann-Gibb [21].

Most of these few studies were conducted over 15 years ago and none differentiated between women without cancer and women who already had a breast or ovarian cancer diagnosis. Therefore, the present study aimed to gain greater insight into the associations of illness representations, coping, and psychological health in women with a *BRCA1/2* mutation. More specifically, we sought to (a) assess cognitive and emotional illness representations and coping strategies in *BRCA1/2* mutation carriers, (b) analyze the association between illness representations, coping strategies and psychological distress (cancer worry and depressive symptoms), and (c) explore whether women who already had a breast or ovarian cancer diagnosis differ from women with no former cancer diagnosis in terms of illness representations, coping strategies and psychological distress. We used the CSM [14,21,22] to develop a conceptual model (see Figure 1) for our data analysis.

## 2. Materials and Methods

### 2.1. Procedures and Participants

This cross-sectional, observational, mono-center study was conducted at the Center of Hereditary Breast and Ovarian Cancer at Charité-Universitaetsmedizin Berlin, which is part of the German Consortium Hereditary Breast and Ovarian Cancer (GC-HBOC). Ethical approval was obtained from Charité-Universitaetsmedizin Berlin (EA1/222/15). The study is described in more detail in Speiser et al. [23].

**Figure 2:**
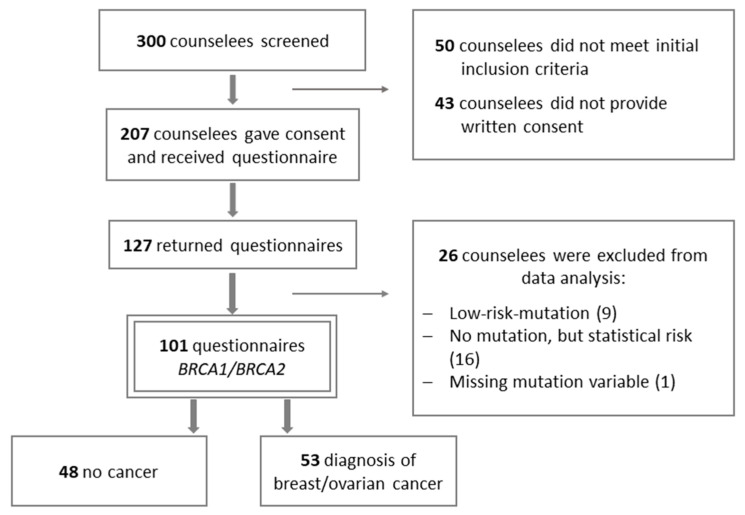
Flowchart of the study population.

Between August 2015 and April 2017, 300 women with a pathogenic BRCA variant, who opted for additional optional counseling, were screened for our initial inclusion criteria. A total of 250 women who met the initial inclusion criteria were invited to participate in the study. Of these, 207 women provided written consent and were subsequently mailed self-report-questionnaires. A total of 127 women returned the questionnaire, of which 26 were excluded from data analysis (see Figure 2). The final inclusion criteria for study participants were women between the age of 18 and 70 years and the detection of a pathogenic mutation in the *BRCA1/2* gene. The exclusion criteria were insufficient German language skills. In total, 101 counselees with the *BRCA1/2* mutation, with an average time of mutation diagnosis of 14.2 months prior to the study, were included in the analysis.

### 2.2. Instrumentation

Sociodemographic variables (age, marital status, presence of children, level of education, occupation status) and clinical variables (history of cancer, gene test result, time since mutation test diagnosis) were assessed with self-report questionnaires. *Illness representations* of breast and ovarian cancer risk were assessed by the Brief Illness Perception Questionnaire (BIPQ)—German version [24]. Scales were modified for use with women at high risk for breast and ovarian cancer to measure cancer risk perception. In total, six of the eight 0–10 point scales were included, measuring the following dimensions of illness perception: (1) consequences, which represents the expected somatic and psychosocial effects of the health threat; (2) treatment control, which refers to the beliefs about the effectiveness of the prevention measures in curing or controlling the health threat; (3) personal control, which reflects the belief in personal abilities to control the health threat; (4) emotional representations, which represents the individual’s (negative) emotional responses associated with the health threat; (5) concern, which refers to how much the person worries about his/her health threat; (6) coherence, which refers to how well the person understands his/her health threat. *Coping strategies* were assessed using four subscales of the Brief COPE—German version [25]: positive reframing, instrumental support, denial, and active coping. Each scale comprises of 2 items with a 4 point-scale, that rate the extent to which individuals have used specific coping efforts in dealing with the breast and ovarian cancer risk. *Cancer worry* was assessed by employing the cancer worry scale (CWS) [26]—German translation [27]. The four items with a 4 point-scale were modified for use in women at high risk for breast and ovarian cancer to measure the degree of worry about developing breast or ovarian cancer. No clinical cut-offs were currently available. *Depressive symptoms* were assessed by applying the Brief Patient Health Questionnaire PHQ-9—German version [28]. The nine-item questionnaire assesses the presence of major depressive disorder using modified DSM-IV criteria with a 4-point scale.

### 2.3. Data Analysis

Demographic and clinical characteristics, illness perceptions, and coping strategies are displayed for the entire sample and for women with and without breast cancer separately. Group differences were analyzed using the *t*-test or Mann–Whitney U test. Relationships between illness representations, coping strategies and cancer worry, or depressive symptoms were calculated with Pearson’s correlation coefficients for continuous variables and Spearman-Rho for ordinal variables. We explored the associations between potential predictors (demographic, clinical, illness perception, and coping variables) and cancer worry or depressive symptoms as outcome variables. Variables with r ≥ 0.20 were included in the subsequent regression analysis. According to Tabachnick and Fidell [29], we used hierarchical stepwise regression to analyze the amount of variance each dimension of the CSM contributed to predicting the criterions cancer worry and depressive symptoms, respectively. Following our conceptual model (see Figure 1), we entered demographic and clinical variables in the first step, followed by illness perceptions in the second step, and coping variables in the third step. We reviewed the models by assessing linearity, homoscedasticity and independence of residuals [30]. We assessed multicollinearity with the variance inflation factor (VIF), which was in all cases below the recommended threshold of <10 [30]. Data processing was performed with the Statistical Package for the Social Sciences (SPSS 24.0).

## 3. Results

### 3.1. Sample Characteristics

Of the 101 participating women included in the analysis, the mean age was 43.4 years (*SD* = 10.9). Most women were partnered (79.2%) and had children (69.3%). A total of 60.4% had a high school degree and 72.3% of the women were employed.

About two thirds of the sample had a *BRCA1* mutation (61.4%) and one third had a *BRCA2* mutation (38.6%). The mean time since detection of the gene mutation was 14.2 years (*SD* = 12.6). The sample was nearly evenly distributed into women who already had a breast or ovarian cancer diagnosis (52.5%) and women with no former diagnosis of breast or ovarian cancer (47.5%). The mean time since cancer diagnosis was 62.1 months (*SD* = 62.6). An overview of the demographic and clinical characteristics of the sample is provided in Table 1.

### 3.2. Illness Perceptions, Coping Strategies, and Psychological Distress

Women with a former cancer diagnosis perceived the consequences of breast or ovarian cancer to be more severe, had more concerns, had more (negative) emotional representations associated with breast or ovarian cancer, and used active coping strategies more often (Table 2). These women also experienced higher levels of cancer worry and more depressive symptoms.

Perceiving more concern, more consequences, and more (negative) emotional representations was significantly associated with active coping and denial (Table 3). Perceptions of more treatment control was significantly related to seeking more social support, active coping, and positive reframing.

### 3.3. Hierarchical Regression Analyses

The correlations between demographic characteristics, clinical characteristics, illness representations, and coping strategies with the criterion variables cancer worry and depressive symptoms can be seen in Table 4. 

Hierarchical stepwise regression was employed to show whether illness representations (step 2) and coping strategies (step 3) improved the prediction of cancer worry and depressive symptoms beyond that explained by differences in demographic and clinical characteristics (step 1). Table 5 displays the unstandardized regression coefficients (*B*) and their standard errors (*SE B*), the standardized regression coefficients (*β*) and their significance value, and the *R*^2^ for the initial model and the change in *R*^2^ (denoted as #x394;*R*^2^) for each subsequent step of the model.

#### 3.3.1. Cancer Worry

After step 1, with demographic characteristics in the equation, *R*^2^ = 0.10, with *F* (2, 95) = 5.37, *p* < 0.01. Only occupation (*β* = 0.24, *p* < 0.05) contributed independently. After step 2, with illness perceptions added to the prediction of cancer worry, *R*^2^ = 0.60, with *F* (6, 91) = 22.50, *p* < 0.01. Two illness representation variables, consequences (*β* = 0.30, *p* < 0.05) and concern (*β* = 0.37, *p* < 0.01) contributed independently. After step 3, with coping strategies added to the prediction of cancer worry, *R*^2^ = 0.60, with *F* (7, 90) = 19.24, *p* < 0.01. The addition of coping strategies did not reliably improve *R*^2^. Nevertheless, two illness representation variables, consequences (*β* = 0.29, *p* < 0.01) and concern (*β* = 0.37, *p* < 0.01), contributed independently. This pattern of results suggests that about 10% of the variability in cancer worry was predicted by demographic characteristics. Illness perceptions contributed considerably to predicting cancer worry, accounting for 50% of the variability in cancer worry. Coping strategies added no further prediction.

#### 3.3.2. Depressive Symptoms

After step 1, with clinical characteristics (prophylactic operations) in the equation, *R*^2^ = 0.07, with *F* (1, 94) = 6.56, *p* < 0.05. After step 2, with illness perceptions added to the prediction of depressive symptoms, *R*^2^ = 0.51, with *F* (5, 90) = 18.73, *p* < 0.01. The variable prophylactic operations (*β* = −0.16, *p* < 0.05) and two illness representation variables, illness coherence (*β* = −0.18, *p* < 0.05) and emotional representation (*β* = 0.59, *p* < 0.01) contributed independently. After step 3, with coping strategies added to the prediction of depressive symptoms, *R*^2^ = 0.52, with *F* (7, 88) = 13.31, *p* < 0.01. The addition of coping strategies did not reliably improve *R*^2^. Only two illness representation variables, illness coherence (*β* = −0.17, *p* < 0.05) and emotional representation (*β* = 0.60, *p* < 0.01) contributed independently. This pattern of results suggests that about 7% of the variability in depressive symptoms was predicted by clinical characteristics. Illness perceptions contributed considerably to predicting depressive symptoms, accounting for 45% of the variability. Coping strategies added no further prediction.

## 4. Discussion

This study aimed to examine the relationship among illness representations, coping strategies and psychological distress (i.e., cancer worry and depressive symptoms) in *BRCA1/2* mutation carriers. Our findings support several assumptions of the CSM [13] applied to women with a *BRCA1/2* mutation, illustrating how these individuals cope with an increased breast and ovarian cancer risk. 

Our findings confirm the CSM’s assumption that illness representations are significantly related to coping strategies. Women who perceive more consequences, more concern about their mutation, more negative emotions (i.e., higher emotional representations), and higher personal and treatment control applied significantly more active coping strategies. Interestingly, perceiving more consequences, more concern, and more negative emotions was also significantly related to using more denial as a coping strategy, which may reflect the urge to do both: proactively address the health threat while avoiding strong emotions associated with high concern and severe consequences. This finding has been described by Krohne and colleagues in the “Model of Coping Modes” [31]. It postulates that individuals might employ increased vigilance and, simultaneously, increased avoidance when confronted with a stressor. Those with this “fluctuating coping mode”, which is also referred to as “high anxiety”, may be threatened by both the uncertainty and the emotional arousal of (health) threatening situations. As it is not possible to cope with both uncertainty and emotional arousal at the same time (e.g., observe the stressor and at the same time ignore it), these individuals might exhibit fluctuating coping strategies [31], as we have seen in our population. 

Our results, in line with other studies using the CSM [16,18,21,22], indicate that illness representations play an important role in psychological distress. This is especially true for cancer worry, as illness representations account for 50% of its variability. In particular, perceiving severe consequences and having strong concerns contributed significantly to cancer worry in our final regression model. This finding is not surprising, given the personal experiences with cancer faced by many families with *BRCA1/2* mutations [7]. These experiences and memories likely shape a high level of concern and the perception of severe consequences associated with the disease. It is conceivable that this perception evokes the fear of developing cancer.

The distinction between women who already had a cancer diagnosis and women with no former diagnosis was unique in this study. Women previously diagnosed with breast or ovarian cancer perceived the consequences of the health threat to be more severe, had more concerns, more negative emotions associated with the health threat, and used more active coping strategies than women with no former diagnosis of breast or ovarian cancer. Women who already had a cancer diagnosis also showed significantly worse psychological distress. This is reflected in the overall high incidences of depressive symptoms and anxiety in breast [32] and ovarian cancer patients [33].

### 4.1. Study Limitations

The current study has some limitations. (1) The process of adaptation to living with *BRCA1/2* gene mutation evolves with time, involving new sources of information in the form of medical and genetic diagnoses, family circumstances or media coverage (“the Angelina Jolie Effect”) [34]. However, the cross-sectional design of this study does not allow for the analysis of illness perceptions, coping strategies and psychological health outcomes over time. Moreover, the relationships shown in such a cross-sectional study cannot be interpreted causally. Longitudinal studies are needed to assess whether illness representations and coping strategies transpire as causal factors in psychological distress outcomes over time. (2) Due to the “Gendiagnostikgesetz”, the German genetic diagnostics law, which guarantees an individual the “right not to know” and not to be informed about clinically relevant findings, the study population may have been relatively well informed, particularly interested and motivated to deal with their situation. There may be a self-selection of individuals choosing to undergo genetic analysis, who “have the ability to tolerate anxiety and to persevere in the face of adversity” [35] or who believe they are able to cope with the situation [36]. This could limit the generalizability of the results.

### 4.2. Clinical Implications

Given the significance of the associations between illness representations and cancer worry, we suggest that physicians involved in counseling *BRCA1/2* mutation carriers be particularly attentive to women with perceptions of severe consequences and concerns about their mutation, or without a coherent understanding of the health threat. Psychological interventions should address both cognitive and emotional illness representations. Genetic counseling should focus on cognitive representations in order to locate catastrophic, incoherent, and unrealistic perceptions of the health threat and gain a more helpful view. There is evidence that challenging maladaptive cognitive representations while simultaneously forming more adaptive representations can lead to enhanced psychosocial outcomes [37].

Many women with a *BRCA1/2* mutation overestimate their risk of developing cancer in the coming years [23]. Physicians should not only inform mutation carriers about different management options available, but also provide risk information in the most comprehensible and transparent manner possible. Research has also suggested genetic counseling should be tailored to the individual and become more interactive [38]. It is therefore important to assess illness representations, risk perception and other relevant factors before providing personalized risk information in the form of 5- and 10-year risk estimates [39]. Women could then be asked to express their ideas and feelings about the risks in their own words, in order to detect possible inaccuracies in their interpretation of the given information [38].

In addition, it may be helpful to offer a place to address fears, anger or feelings of hopelessness in order to improve emotional wellbeing. There is evidence of positive effects through supportive–expressive group therapy, in which women with *BRCA1/2* mutations were encouraged to express their emotions, received the opportunity to confront newly formed existential challenges and were able to create new meaning in life [40]. 

Future research should focus on clinical interventions for both cognitive and emotional illness representations. On the basis of our results, interventions should specifically target perceptions of severe consequences, high concern about the mutation, negative emotional illness representations and low coherence of the mutation in order to reduce potential psychological distress. As noted previously, illness representations evolve over time. Future research should seek to investigate illness representations and coping strategies repeatedly in longitudinal studies, in order to gain more profound insight into the adjustment process over time.

## 5. Conclusions

Our findings illuminate the importance of cognitive and emotional illness representations in understanding individual responses to living with a *BRCA1/2* mutation. In particular, perceiving severe consequences, having more concerns and perceiving the mutation as being incoherent are significantly associated with cancer worry and depressive symptoms. Women with a former diagnosis of breast or ovarian cancer show patterns of illness representations that are potentially more distressing than women without cancer. These findings will become even more relevant in the future, as rapidly advancing technology makes testing for cancer susceptibility genes more widely available [41]. Physicians involved in counseling should pay attention to both cognitive and emotional illness representations to detect maladaptive thoughts associated with the mutation, address negative emotions, and stimulate adaptive coping strategies.

## Figures and Tables

**Table 1:** Demographic and clinical characteristics of the entire study population and according to previous diagnosis of cancer.

	Entire Sample (*N* = 101)	Diagnosis of Breast/Ovarian Cancer (*n* = 53)	No Breast/Ovarian Cancer (*n* = 48)
**Demographic characteristics**			
**Age (years), *M (SD)***	43.4 (10.9)	46.7 (9.6)	40.0 (11.2)
**Partnership, *n* (%)**			
Living with a partner	80 (79.2%)	40 (75.5%)	40 (83.3%)
Living without a partner	21 (20.8%)	13 (24.5%)	8 (16.7%)
**Presence of children, *n (%)***	70 (69.3%)	38 (71.7%)	32 (66.7%)
**Level of education, *n (%)***			
High school degree	61 (60.4%)	28 (52.8%)	33 (68.8%)
Secondary school	40 (39.6%)	25 (47.2%)	15 (31.3%)
**Occupation status, *n (%)***			
Employed	73 (72.3%)	35 (66.0%)	38 (80.9%)
Unemployed	13 (12.9%)	7 (13.2%)	6 (12.5%)
Retired	14 (13.9%)	11 (20.8%)	3 (6.3%)
**Clinical Characteristics**			
**Prophylactic surgery_,_*n (%)***			
No prophylactic surgery	46 (45.5%)	17 (32.1%)	29 (60.4%)
Prophylactic mastectomy	13 (12.9%)	9 (17%)	4 (8.3%)
Prophylactic salpingo-oophorectomy	27 (26.7%)	13 (24.5%)	14 (29.2%)
Mastectomy and salpingo- oophorectomy	11 (10.9%)	10 (18.9%)	1 (2.1%)
**History of cancer, *n (%)***			
Breast cancer	-	44 (83.3%)	-
Ovarian cancer	-	5 (9.4%)	-
Months since diagnosis, *M (SD)*	-	62.1 (62.5)	-
**Pathogenic germline variant, n (%)**			
*BRCA1* mutation	62 (61.4%)	35 (66%)	27 (56.3%)
*BRCA2* mutation	39 (38.6%)	18 (34.0%)	21 (43.8%)
Months since mutation analysis, *M (SD)*	14.2 (12.6)	14.3 (11.7)	14.1 (13.6)

**Table 2:** Descriptive statistics for dimensions of illness representations, coping strategies, and psychological distress according to breast cancer diagnosis.

	Entire Sample (*N* = 101)	Diagnosis of Breast/Ovarian Cancer (*n* = 53)	No Breast/Ovarian Cancer (*n* = 48)	95% CI	*p*
**Illness representations** *M (SD)*					
Consequences	4.8 (2.6)	6.3 (2.5)	5.5 (2.6)	−2.50 to −0.49	**0.004**
Illness coherence	7.3 (2.0)	7.0 (2.4)	7.2 (2.2)	−0.59 to 1.13	0.532
Concern	5.9 (2.6)	6.5 (2.3)	5.3 (2.9)	−2.21 to −0.16	**0.023**
Emotional representations	4.8 (2.9)	6.3 (2.6)	5.6 (2.9)	−2.68 to −0.50	**0.005**
Personal control	5.4 (2.4)	5.8 (2.3)	5.6 (2.4)	−1.29 to 0.58	0.453
Treatment control	6.8 (2.0)	6.3 (2.1)	6.5 (2.1)	−0.32 to 1.30	0.231
**Coping strategies** *M (SD)*					
Active coping	1.5 (1.1)	1.9 (1.1)	1.2 (1.0)	−1.06 to −0.23	**0.003**
Social support seeking (instrumental)	1.3 (0.9)	1.3 (1.0)	1.3 (0.9)	−0.45 to 0.28	0.645
Denial	0.4 (0.7)	0.4 (0.7)	0.4 (0.7)	−0.33 to 0.23	0.726
Positive reframing	1.5 (0.9)	1.6 (0.9)	1.3 (1.0)	−0.58 to 0.16	0.254
**Psychological distress** *M (SD)*					
Cancer worry	1.7 (0.8)	1.9 (0.7)	1.5 (0.9)	−0.78 to −0.15	**0.005**
Depressive symptoms	0.7 (0.5)	0.8 (0.5)	0.5 (0.4)	−0.45 to −0.08	**0.006**

Group differences analyzed using *t*-test.

**Table 3:** Associations of illness representations and coping strategies.

	Active Coping	Social Support Seeking	Denial	Positive Reframing
1. Consequences	0.37 *	0.16	0.23 *	0.06
2. Illness coherence	−0.09	0.13	−0.06	−0.09
3. Concern	0.29 *	0.07	0.26 *	−0.18
4. Emotional representations	0.28 *	0.17	0.30 *	−0.09
5. Personal control	0.26 *	0.15	0.15	0.21 *
6. Treatment control	0.23 *	0.28 *	0.06	0.27 *

Correlations calculated with Pearson. * *p* < 0.05.

**Table 4:** Associations of sociodemographic variables, clinical characteristics, illness representations, and coping with cancer worry and depressive symptoms.

	Cancer Worry	Depressive Symptoms ^b^
**Demographic Characteristics**		
Age	0.05	0.16
Marital status ^a^	0.06	0.15
Presence of children ^a^	0.20 *	0.08
Level of education ^a^	0.17	0.19
Occupation status ^a^	0.30 *	0.19
**Clinical characteristics**		
Subgroups operations ^a^	0.21	0.29 *
History of cancer ^a^	0.28	0.29 **
Months since cancer diagnosis	0.09	0.33 *
Mutation diagnosis ^a^	0.14	0.11
Months since mutation analysis	−0.16	0.08
**Illness representations**		
Consequences	0.68 **	0.46 **
Illness coherence	−0.23 *	−0.30 **
Concern	0.70 **	0.47 **
Emotional representations	0.64 **	0.67 **
Personal control	0.05	0.10
Treatment control	−0.05	−0.16
**Coping strategies**		
Active coping	0.31 **	0.29 **
Social support seeking	0.08	0.14
Denial	0.14	0.24 *
Positive reframing	0.08	0.05

Correlations calculated with Pearson for metric scales. * *p* < 0.05; ** *p* < 0.01; ^a^ correlations calculated with Eta and ANOVA for nominal-metric scales; ^b^ square root transformed; * *p* < 0.05; ** *p* < 0.01.

**Table 5:** Multiple stepwise regression analysis (method enter, using three steps), prediction of cancer worry and depressive symptoms by demographic variables, the illness representation dimensions and coping strategies.

Cancer Worry	*B*	*SE B*	*β*
Step 1			
Presence of children	0.27	0.12	0.15
Occupation	0.28	0.18	0.24 *
Step 2			
Presence of children	0.11	0.13	0.06
Occupation	0.09	0.09	0.08
Consequences	0.09	0.03	0.30 *
Coherence	−0.05	0.03	−0.12
Concern	0.12	0.03	0.37 **
Emotional representation	0.03	0.03	0.12
Step 3			
Presence of children	0.11	0.13	0.81
Occupation	0.09	0.09	1.03
Consequences	0.09	0.03	0.29 **
Coherence	−0.05	0.03	−0.12
Concern	0.12	0.03	0.37 **
Emotional representation	0.03	0.03	0.12
Active coping	0.04	0.05	0.05
*R*^2^ = 0.10 for block 1; Δ*R*^2^ = 0.50 for block 2; Δ*R*^2^ = 0.00 for block 3. * *p* < 0.05; ** *p* < 0.01			
**Depressive Symptoms**	***B***	***SE B***	***β***
Step 1			
Prophylactic operations	0.04	0.05	0.26 *
Step 2			
Prophylactic operations	0.02	0.01	0.16 *
Consequences	−0.01	0.02	−0.04
Coherence	−0.03	0.01	−0.18 *
Concern	0.01	0.02	0.08
Emotional representation	0.07	0.01	0.59 **
Step 3			
Prophylactic operations	0.02	0.01	1.13
Consequences	−0.01	0.02	−0.06
Coherence	−0.03	0.01	−0.17 *
Concern	0.01	0.02	0.08
Emotional representation	0.07	0.02	0.60 **
Active coping	0.02	0.03	0.07
Denial coping	−0.01	0.04	−0.01
*R*^2^ = 0.07 for block 1; Δ*R*^2^ = 0.45 for block 2, Δ*R*^2^ = 0.00 for block 3. * *p* < 0.05; ** *p* < 0.01			

## Data Availability

The data that support the findings of this study are available on request from the corresponding author. The data are not publicly available due to ethical restrictions.
